# Post-traumatic stress disorder and its associated factors among people who experienced traumatic events in east African countries, 2020: a protocol for systematic review and meta-analysis

**DOI:** 10.1186/s12991-020-00324-0

**Published:** 2021-01-09

**Authors:** Mengesha Srahbzu Biresaw, Enguday Tirfeneh Gebeyehu

**Affiliations:** 1 Department of Psychiatry, College of Medicine and Health Sciences, University of Gondar, Gondar, Ethiopia; 2 Department of Psychiatry, College of Health Sciences, Aksum University, Aksum, Ethiopia

**Keywords:** Post-traumatic stress disorder, Adults, Ethiopia

## Abstract

**Background:**

Post-traumatic stress disorder (PTSD) is the most commonly reported mental health consequence following disasters and traumatic events, either natural or man-made. Nothing is written regarding its pooled prevalence and pooled estimate of factors. Therefore, this study aimed to determine the pooled prevalence of PTSD and estimate the pooled effect of associated factors.

**Methods:**

An English version of published articles will be retrieved using the following; PubMed/Medline, Africa-wides, Science Direct, Cochrane Library, Global Health, Google Scholar, EMBASE, and psycINFO. Research reports will be searched from October 10/2020 to November 10/2020. The research reports quality will be assessed using the Newcastle–Ottawa Scale. Relevant information from the searched research reports will be extracted in a Microsoft Excel format. After extraction, the data will be imported to STATA version 14.0 for analysis. An appropriate guideline for a systematic review and meta-analysis report will be used, i.e. the Preferred Reporting Items for Systematic reviews and Meta-Analyses. A random-effects meta-analysis model will be used to estimate the Der Simonian and Laird’s pooled prevalence of PTSD and its associated factors.

**Discussion:**

This study aims to determine the pooled prevalence of PTSD and estimate the pooled effect of associated factors. Several kinds of research have reported the increasing magnitude of PTSD and its determinants in a different population. This might be due to reasons, such as little attention being given to the issue. Therefore, this study will try to fill this gap by giving new evidence-based results to attract policymakers’ attention.

## Background

According to the Diagnostic and Statistical Manual of Mental Disorders 5th edition (DSM-5), Post-Traumatic Stress Disorder (PTSD) is diagnosed in people who have experienced traumatic events in their day-to-day activities directly or indirectly. Traumatic events include being exposed to actual or threatened death, serious injury, being diagnosed with a life-threatening disease, torture, sudden unexpected death of a loved one, and military combat or sexual violence [1]. PTSD manifestations are found to be common among people who have experienced traumatic events when compared to their counterparts [2].

PTSD is the most commonly reported mental health consequence particularly for both man-made events like disasters and traumatic events [3]. The burden of untreated PTSD is enormous since it causes prolonged morbidity, impairment in day-to-day activities, and poor quality of life in all dimensions including health, productivity, and social interaction [1, 4, 5]. It affects all groups of the population who have been experienced stressful life events regardless of the individuals’ characteristics including gender, age, race [6, 7].

PTSD contributes to a substantial percentage of the burden of disability both in the developed and developing world [8, 9]. According to the World Mental Health Survey conducted recently, PTSD was among the most frequently occurring and debilitating psychiatric disorders reported to cover 54.8% and 41.2% disability in developed and developing countries, respectively [10].

Globally, around 8 million people developed PTSD per single year [11]. Stress-related disorders including PTSD were projected to be the second leading cause of disability by the year 2020 in a survey by the World Health Organization to estimate the burden of disease [12]. PTSD has been reported to account for about 0.4% of the total years lived with disability and it has been estimated to increase to 0.6% globally [13]. The global economic burden of stress-related mental illness is expected to rise in the coming decade [12].

Several studies have been conducted to report the prevalence of PTSD and its determinants among people exposed to traumatic life events worldwide. The lifetime prevalence of PTSD in the United States of America (USA) was shown to be 8% in the general population [14]. A study from Iran reported the prevalence of PTSD among commercial motor vehicle drivers to be 19.2% [15]. Another study from Korea reported a one-year prevalence of PTSD among subway drivers to be 5.6% [16]. A study from Israel among people exposed to terrorism showed that the prevalence of PTSD was 9.4% [17].

In a large epidemiological survey conducted between 1997 and 1999, among survivors of war or mass violence in low-income countries, the prevalence rate of assessed PTSD was 37.4% in Algeria, 28.4% in Cambodia, 15.8% in Ethiopia, and 17.8% in Gaza. In this survey, conflict-related trauma was a risk factor for PTSD that was present in all four samples. Torture was abundantly reported in all samples except Cambodia. Psychiatric history and current illness were risk factors in Cambodia and Ethiopia. The poor quality of the camp was associated with PTSD in Algeria and Gaza. Daily hassles were associated with PTSD in Algeria. Youth domestic stress, death or separations in the family and alcohol abuse in parents were associated with PTSD in Cambodia [18].

There have been a number of studies conducted in Ethiopia which have demonstrated the variety of traumas experienced and their associated factors. A cross-sectional study conducted at Addis Ababa; Ethiopia reported the prevalence of PTSD to be 22.8% among survivors of a road traffic accident. In these study factors, such as being female, having poor social support, duration since the accident (1–3 months), and having depression were reported as significantly associated with PTSD [19]. In another cross-sectional study conducted in southwest Ethiopia, the prevalence of PTSD was reported to be 12.6%. Factors, such as a history of a near-miss road traffic crash, depression, and high cannabis use, were reported as having a significant association with PTSD [20].

Another study conducted among landslide survivors in Ethiopia showed the prevalence of PTSD to be 37.3%. In this study, factors including female sex, divorce, sustained physical injury, having a history of mental illness, family history of mental illness, poor social support, and high perceived stress were reported as significantly associated with PTSD [21].

PTSD has been investigated in different studies conducted in east African countries. Although overall there is a high prevalence of PTSD and mental health co-morbidities following traumatic events, the reported prevalence varies greatly in different studies conducted. There is also a great variation in the factors associated with PTSD. Therefore, the main goal of this systematic review and meta-analysis study will be to determine the pooled prevalence of PTSD and associated factors among people who have experienced traumatic events in east African countries.

## Research questions about the pooled prevalence and associated factors of PTSD

This study plans to examine the pooled prevalence of PTSD among people who experienced traumatic events in east African countries in 2020. Literature has indicated different prevalence of PTSD among different groups of people exposed to traumatic events, this systematic review and meta-analysis study will try to examine the degree of variation in the prevalence of PTSD between countries and population groups. The source of heterogeneity will also be identified by sorting the data based on their methodologies.

This systematic review and meta-analysis are expected to determine the pooled effect estimates of factors associated with PTSD among people who experienced traumatic events in East African countries, 2020.

## Methods

### Identification and selection of studies

A systematic review of published English language research which reports the prevalence and associated factors of PTSD among people who experienced traumatic events in east African countries will be considered. A search of research reports will be made by the following databases: PubMed/Medline, Africa-wides, science direct, Cochrane Library, Global Health, Google Scholar, EMBASE, and psycINFO. Research reports from October 10 to November 10/2020 will be included. The primary search items will be (prevalence OR epidemiology OR magnitude) AND (post-traumatic stress disorder OR PTSD OR acute stress disorder) AND (associated factors OR predictors OR risk factors) AND (Ethiopia) AND (Kenya) AND (Uganda) AND (Tanzania). An appropriate guideline for a systematic review and meta-analysis report will be used, i.e. the Preferred Reporting Items for Systematic reviews and Meta-Analyses (PRISMA-p) [22].

## Eligibility criteria

### Inclusion criteria

All relevant research reports which will be available on the search until November 10/2020 will be included based on the following inclusion criteria.

1. a study which reported the prevalence of PTSD
2. conducted in East African countries
3. a study which applied probable sampling technique

### Exclusion criteria

The following studies will be excluded.

1. Experimental, qualitative, and psychometric studies
2. Studies which are not written in English
3. Studies which cannot be fully accessed after a request is made from their author by email

### Data extraction

MS and ET will independently extract all the necessary data using a standardized data extraction format. These data extraction formats will be prepared to include the following items: the first author, publication year, region of the study conducted, sample size, a screening tool used to detect PTSD, response rate, the prevalence of PTSD, and associated factors with PTSD. Cross-checking will be done by MS and ET following searches. There will be further discussion to achieve consensus and double extraction will be made to solve any disagreements between the two authors. If required, we will contact the original authors for more clarification. We will apply kappa statistics to indicate the difference between observed and expected agreements between authors, at random or by chance only. We will also conduct a sensitivity analysis to assess the robustness of meta-analytic results.

### Outcome measurements

We have two objectives in this systematic review and meta-analysis study. These are to determine the pooled prevalence of PTSD among people exposed to traumatic events in East African countries and to estimate the pooled effects of associated factors with PTSD among people who experienced traumatic events in these countries. The pooled prevalence of PTSD will be calculated using STATA version 14.0. The pooled effect estimate of associated factors with PTSD will be calculated. The odds ratio will be prepared from the searched research reports using two by two tables.

### Quality assessment

The quality of the research reports included in this systematic review and meta-analysis study will be assessed using the Newcastle–Ottawa Scale for cross-sectional studies quality assessment [23]. Articles that meet the minimum requirements, i.e. at least 50% of the quality assessment criteria and high quality meaning articles that score 6 out of 10 scales will be included for analysis.

### Statistical procedure

The relevant information from the searched research reports will be extracted in a Microsoft Excel format. The data will then be imported to STATA version 14.0 for analysis. The characteristics of the original articles will be presented using texts, tables, and forest plots. The standard error of prevalence for each original article will be calculated using the binomial distribution formula. The prevalence of the reported researches will be checked for heterogeneity using a heterogeneity *χ*^2^ test and *I*^2^ test. A random-effects meta-analysis model will be used to estimate the Der Simonian and Laird’s pooled prevalence of alcohol use and associated factors. Publication bias will be checked by performing Egger’s correlation and Begg’s regression intercept tests at a 5% significant level. If there is evidence of publication bias in our analysis, we will perform a Duval and Tweedie non-parametric 'trim and fill' analysis to formalize the use of funnel plot, estimate the number and outcome of missing studies, and adjust for theoretically missing studies.

Subgroup analysis will be conducted to identify the impact of variables in a particular group for the prediction of the pooled prevalence of PTSD. We will perform a leave-one-out sensitivity analysis to check how the predicted pooled prevalence of PTSD and its conclusion alters when a single study result is removed from the analysis. We will use the sensitivity analysis result to identify the possible source of heterogeneity if we detect it during analysis.

The prediction interval will be computed to reflect the variation of pooled prevalence of PTSD in different setting. We will make recommendations for further research.

## Discussion

This study aims to determine the pooled prevalence of PTSD and estimate the pooled effect of associated factors. Several research studies using differing methods have reported the magnitude of PTSD and its determinant factors in a varying population, and shown that the prevalence is increasing. This might be due to reasons, such as little attention being given to the issue. This study will try to fill this gap by giving new evidence-based results to attract policymakers’ attention. The results of this study will hopefully identify questions for future research.

This study will be reported using a standardized reporting guideline for systematic review and meta-analysis of observational studies that is the Preferred Reporting Items for Systematic reviews and Meta-Analyses (PRISMA-P) checklist [22].

We can mention that this study’s plan to include all observational studies for systematic review and meta-analysis is a strength. The strength of this study is also the independent research report searching, selection and data extraction pursued by two independent reviewers. This study will be limited if a high level of heterogeneity is observed during analysis.

## Data Availability

Not applicable.

## Abbreviations

PRISMA-P: Preferred Reporting Items for Systematic Reviews and Meta-analysis
PTSD: Posttraumatic stress disorder
USA: United States of America
